# Regulatory B Cells in Seropositive Myasthenia Gravis versus Healthy Controls

**DOI:** 10.3389/fneur.2017.00043

**Published:** 2017-02-20

**Authors:** Md Rezaul Karim, Hong-Yan Zhang, Jiang Yuan, Qiang Sun, Yun-Fu Wang

**Affiliations:** ^1^Department of Neurology, Taihe Hospital of Hubei University of Medicine, Shiyan, China

**Keywords:** regulatory B cells, CD19^+^CD5^+^CD1d^+^ Bregs, flow cytometry, MG, ELISA, IL-10, TGF-β1

## Abstract

**Objective:**

To find out if the failure in immunotolerance of myasthenia gravis (MG) is a possible aspect of deduction in Breg cells and to characterize B cell subsets in MG.

**Methods:**

Flow cytometry detection and enzyme-linked immunosorbent assays in peripheral blood films of 10 MG patients and 10 healthy controls (HCs) were performed after isolation of B cells. The CD19^+^CD5^+^CD1d^+^ Breg cells percentages were measured to complement a B cell phenotype assay and frequencies of B cell subsets. The clinical outcome measures and immunological variables of patients with MG were compared with HCs.

**Results:**

Patients with MG had relatively lowered percentages of CD19^+^CD5^+^CD1d^+^ Breg cells as compared to HCs. The production of interleukin (IL)-10 and transforming growth factor (TGF)-β1 was relatively lesser in patients with MG than HCs, which were linked with more severe of MG disease status according to Myasthenia Gravis Foundation of America (MGFA) clinical classification. The reduction of cytokine production was more significant for IL-10 than TGF-β1 when compared to HCs.

**Conclusion:**

It has been observed that the reduced number of B cells is able to produce IL-10 in MG patients but lesser than compared to HCs. The Bregs reduction mainly was regarded by the severity of disease status, which was highly significant and also by disease duration which was statistically significant as well. The findings of the measurement of B cell phenotype assay and frequencies of B cell subsets between MGs and HCs give us new ideas to develop B cell-mediated therapies of MG such as (1) isolated B cells of MGs could be cultured with steroids, e.g., dexamethasone *in vitro* to see if it induces the CD19^+^CD5^+^CD1d^+^ Breg cells, (2) it may observe whether induced CD19^+^CD5^+^CD1d^+^ Bregs have higher production of IL-10 and TGF-β1, as both are linked with disease severity, and (3) after completion *in vitro* steps, through further research *in vivo* to observe whether it improves the function of MG disease status.

## Introduction

In most cases of myasthenia gravis (MG), myasthenia results from free-flowing antibodies of bloodstream, which obstructs acetylcholine receptors (AChRs) at the postsynaptic myoneural junction (1). Almost 85% MG patients have free-flowing anti-AChR antibodies (2). Muscle-specific tyrosine kinase (MuSK) autoantibodies (3, 4) are identifiable in 38–47% MG patients those do not possess identifiable antibody to AChR (3, 5, 6). In the course of immune reaction B cells actively shows positive and negative regulatory effects (7). They positively modulate immune reaction by creating antigen-specific antibody and influencing optimal T cell activation (8, 9). Moreover, they negatively modulate immune reaction by immune modulatory cytokines production, which is described in different ranges of mouse models (7, 10–20) are now well established (7, 14, 21). B cells modulatory function in autoimmune diseases was proclaimed by Janeway and colleagues in EAE (17, 22). Bregs presence was subsequently supported by other investigators (13, 22, 23), which shows as of T cell, B cell subsets are also able to influence immunotolerance (7, 21, 22, 24–27). One of the Bregs subsets interleukin (IL)-10 producing B cells (B10 cells) is predominantly found within a phenotypically unique CD1d^hi^CD5^+^CD19^+^ subset (13, 18, 21, 22, 28). The prevalence and characterization of B10 cells that are described in autoimmune and inflammatory diseases involving rheumatoid arthritis (RA), Graves’ disease, and systemic lupus erythematosus (SLE) (29–33). Significantly many studies show that Bregs express decreased and deteriorated characterization (29, 32, 33). Anyhow, B cell subpopulation has not been characterized in MG patients (32). In this research investigation, the presence and dynamics of Breg cells in patients with MG is compared with healthy controls (HCs).

## Bregs: Its Development and Function

Bregs are those immunosuppressive cells that support immunological tolerance. In these recent years, multiple studies in both mice and humans have established that Bregs suppress inflammatory responses primarily *via* the provision of IL-10 (14). These cells regulate the immune system by various mechanisms. The main mechanism is through the production of IL-10, IL-35, and transforming growth factor (TGF)-β1 (34). It is thought that Bregs arise from a common progenitor T2-MZP B cells. These T2-MZP B cells are at an immature point of development and are thought to be autoreactive after interacting with environmental triggers. After T2-MZPs are activated by toll-like receptors on pathogens the first wave of IL-10 is released (14). IL-10 has strong anti-inflammatory effects (35), and it inhibits or suppresses inflammatory responses mediated by T cells. The produced IL-10 by Bregs can repress noxious immune reaction through controlling Th1/Th2 stability and through reducing intrinsic cell-intervened inflammation (36). Bregs also produce another anti-inflammatory cytokine TGF-β (35). Bregs subset that is able to produce TGF-β1 *in vitro* has been determined (37, 38). TGF-β1-producing Bregs subset participates in the initiation of low-dose oral tolerance (38).

## Aims and Objectives

To identify the existence of Bregs and characterization of Bregs in MG in comparison with HCs. To understand the role of Bregs including IL-10 and TGF-β1 secretion in patients with MG in comparison with HCs, which may contribute to new B cell-mediated therapies of MG. CD5^+^CD19^+^CD1d^+^ Breg cells are to be characterized by flow cytometry detection of isolated B cells and the expression level in both MG patients and HCs are to measure. Through an observation with enzyme-linked immunosorbent assay (ELISA), it can be known if the decreased number of Bregs is able to produce IL-10 and TGF-β1 in MG patients.

## Materials and Methods

### Materials and Equipment

Sample blood; anticoagulant; lymphocyte separation medium (LSM); phosphate buffered saline (PBS); buffer; fetal bovine serum (FBS); human antibiotic (HuAB); RPMI 1640 medium; B cell isolation kit II; human IL-10 and TGF-β1 ELISA kit; PerCP-cy™5.5 mouse anti-human CD19; FITC mouse anti-human CD5; PE mouse anti-human CD1d; clinical centrifuges; water bath (37°C); refrigerator; cell culture dishes and flasks; centrifuge tubes; pipettes; hemocytometer; MS column; miniMACS separator; standard ELISA microplate reader; and flow cytometer.

### Standard Protocol Approval, Registration, Patients Consent

The study was carried out in accordance with the recommendation of the Institutional Review Board. This research project was approved by the Hubei University of Medicine, Shiyan, Hubei 442000, China. The research project was specifically reviewed and approved by the evaluation committee of the Hubei University of Medicine. Informed consent was obtained from all MG patients and HCs. Before including, the participants were explained the aim of the research project. Once the verbal consent was understood and agreed, a written consent was then gathered from each of the participants including their legal identification. Those individuals or patients agreed to give the written consent were only included in this research project.

### Study Population and HCs

Patients with MG were involved from Taihe Hospital, Shiyan, Hubei 442000, China. Sample blood was obtained from 10 patients with MG; age between 16 and 58 years. All MG patients had detectable anti-AChR/anti-MuSK antibodies at the time of diagnosis among which seven MG patients had detectable anti-AChR antibodies. Data collection (see Table 1) has taken such as disease duration, medications taken (prednisone, cyclosporine or mycophenolate mofetil, and so on), or thymectomy status. Blood samples were collected in the morning prior taking any medications so that the Bregs were not affected by current medications taken, medications doses, and treatment duration. So that the drugs did not affect the flow cytometry results and the severity of the disease measured by quantitative myasthenia gravis (QMG) scoring and MGFA class. Patients were having the usual adult doses and continued medication when the patient was diagnosed for the first time with MG. The QMG scoring was done according to the QMG test manual by MGFA (39, 40).

**Table 1:** **Data collection sheet of myasthenia gravis patients**.

Sl. No.	Age (years)	Sex (M/F)	Disease time (months)	Meds taken	Thymectomy status	Myasthenia Gravis Foundation of America (MGFA) class	MGFA class on blood draw	Quantitative myasthenia gravis score
01	39	F	9	Pyrid	Not done	Class IIa	Class I	4
02	25	F	24	Pred	Not done	Class IIb	Class IIa	13
03	51	F	120	Pyrid + Pred	Not done	Class IVb	Class IIIb	30
04	51	F	36	Cyclo	Not done	Class IIa	Class IIa	16
05	23	F	4	Pyrid	Not done	Class I	Class I	4
06	22	M	10	None	Done	Class IIIb	Class IIb	24
07	27	F	120	MMF	Not done	Class IVa	Class IIIa	26
08	47	M	12	Pyrid + Aza	Not done	Class IIIa	Class IIb	22
09	16	F	12	Pred	Not done	Class I	Class I	5
10	58	M	4	Pyrid	Not done	Class I	Class I	3

*Pred, prednisone; Cyclo, cyclosporine; Aza, azathioprine; Pyrid, pyridostigmine; MMF, mycophenolate mofetil*.

Blood samples from 10 HCs were obtained from those were not receiving any treatment for any other autoimmune diseases. HCs were matched to the patients with regard to the age, gender, and weight.

### Isolation and Storage of Peripheral Blood Mononuclear Cells (PBMCs)

Peripheral blood was collected in a 20-ml disposable syringe, and anticoagulant was added to the sample blood. The blood sample was first diluted in PBS (Goodbio, Wuhan, China) gently, then according to the manufacturer’s protocol, PBMCs were isolated using LSM (TBD sciences, Tianjin, China) and added culture medium (RPMI 1640 Medium + 10% FBS + 1% HuAB) then transferred to the cell culture flask using a pipette.

### Isolation and Storage of B Cells

The PBMCs were counted using a hemocytometer and determined the cell number. It was then isolated using B cell isolation kit II (Miltenyi Biotec, Auburn, CA, USA) and MS Columns (Miltenyi Biotec, Auburn, CA, USA). Added culture medium (RPMI 1640 Medium + 10% FBS + 1% HuAB) on the isolated B cells then transferred to a cell culture dish using a pipette.

### Flow Cytometry

**Figure 1:**
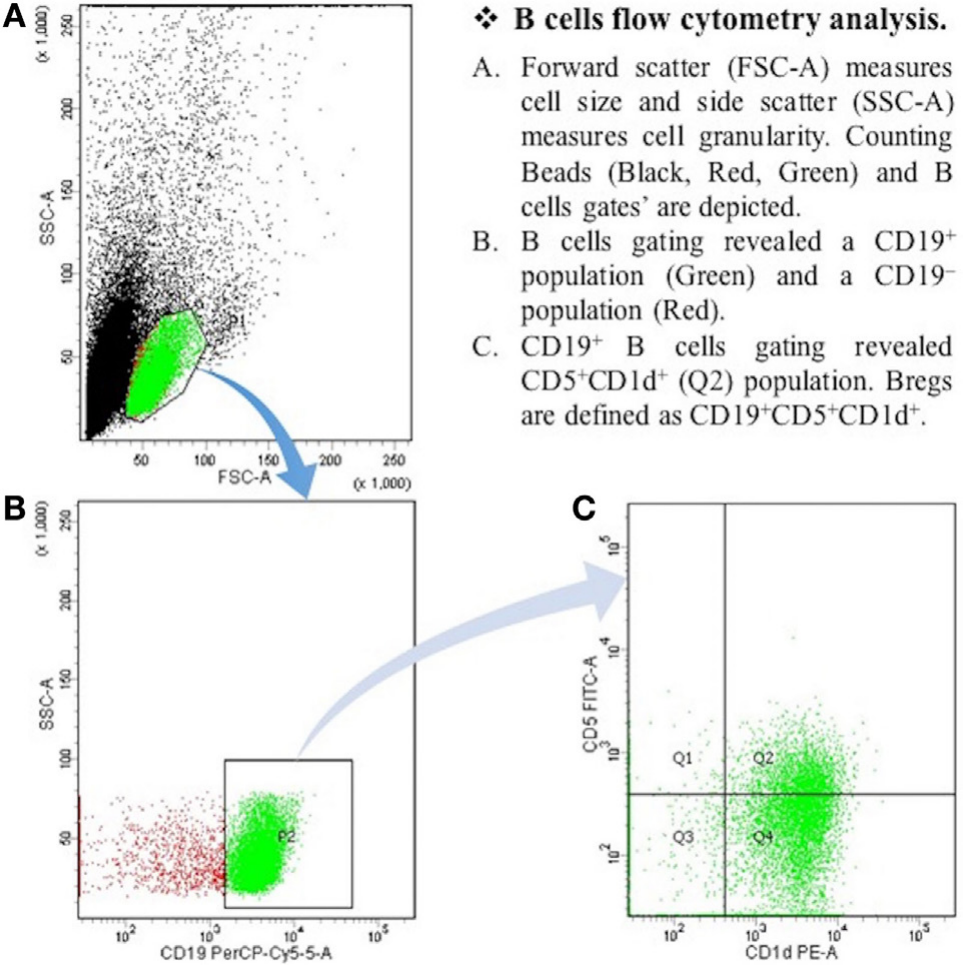
**The expression of CD19^+^CD5^+^CD1d^+^ Bregs in healthy controls**. B cells flow cytometry analysis. **(A)** Forward scatter (FSC-A) measures cell size, and side scatter (SSC-A) measures cell granularity. Counting beads (black, red, and green) and B cells gates are depicted. **(B)** B cells gating revealed a CD19^+^ population (green) and a CD19^−^ population (red). **(C)** CD19^+^ B cells gating revealed CD5^+^CDld^+^ (Q2) population. Bregs are defined as CD19^+^CD5^+^CDld^+^.

**Figure 2:**
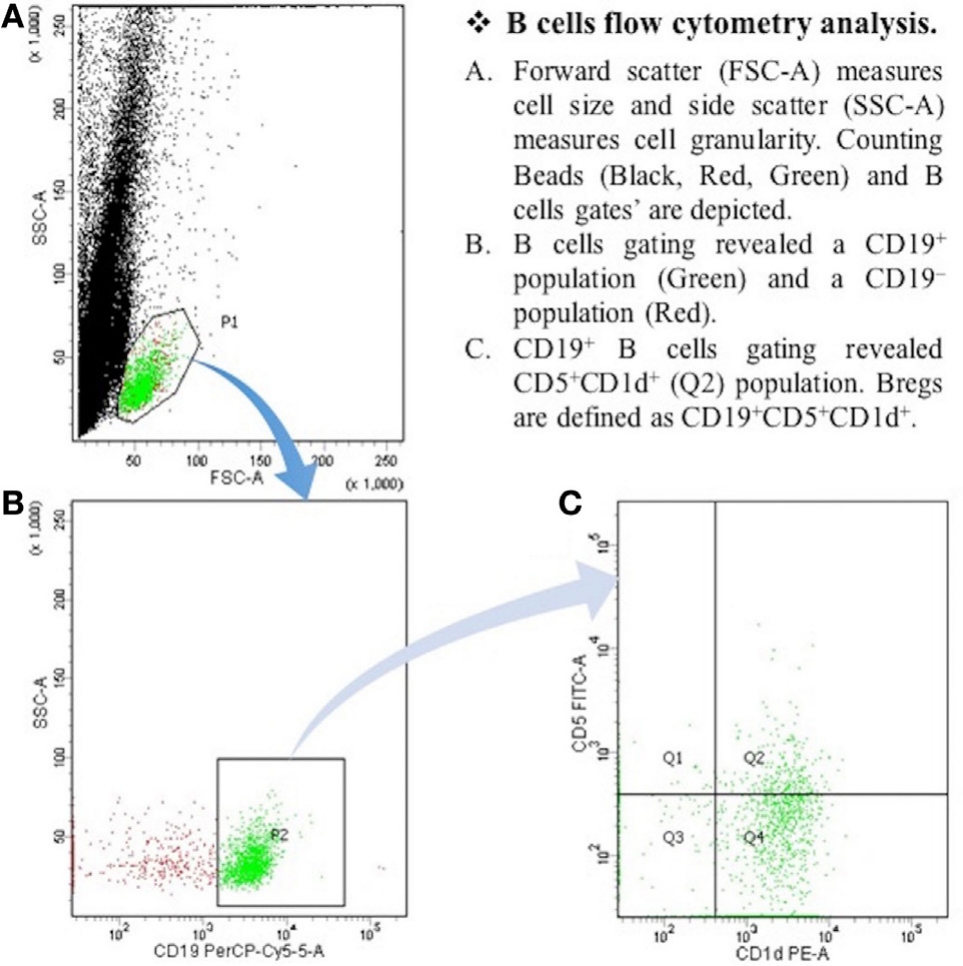
**The expression of CD19^+^CD5^+^CD1d^+^ Bregs in myasthenia gravis patients**. B cells flow cytometry analysis. **(A)** Forward scatter (FSC-A) measures cell size, and side scatter (SSC-A) measures cell granularity. Counting beads (black, red, and green) and B cells gates are depicted. **(B)** B cells gating revealed a CD19^+^ population (green) and a CD19^−^ population (red). **(C)** CD19^+^ B cells gating revealed CD5^+^CDld^+^ (Q2) population. Bregs are defined as CD19^+^CD5^+^CDld^+^.

After the centrifugation and removal of media, cells were surface stained with PerCP-Cy™5.5 mouse anti-human CD19; PE mouse anti-human CD1d; and FITC mouse anti-human CD5 (BD Biosciences, San Diego, CA, USA). After 30 min of incubation in a refrigerator at 4°C, cell suspension was resuspended in PBS and it was then ready for the flow cytometry detection. The samples were read by “BD FACSCantoTM II” flow cytometer (BD Biosciences, San Jose, CA, USA), and data were analyzed by “BD FACSDivaTM V7.0” software (see Figures 1 and 2).

### Enzyme-Linked Immunosorbent Assay

Human IL-10 and human TGF-β1 ELISAs were performed using the human IL-10 ELISA kit and human TGF-β1 ELISA kit (NeoBioscience, Shenzhen, China) according to the manufacturer’s protocols. Absorbency was measured using a standard ELISA microplate reader (Thermo Fisher Scientific, Vantaa, Finland). Unused reagents after the experiment are completed, stored back in a refrigerator to restore temperature.

### Data Analysis and Statistics

Data are shown as (means ± SD) and analyzed by “SPSS V22.0.” The Student’s two-tailed *t*-test was done to identify significant differences between sample means. *P* < 0.05 was considered statistically significant.

## Results and Observation

### Flow Cytometry Detection

#### Bregs Expression in MG and HCs

Reduced CD19^+^CD5^+^CD1d^+^ Bregs expression significantly in B cells of patients with MG (*n* = 10, 19.09 ± 2.61%) as compared to HCs (*n* = 10, 31.42 ± 5.55%) (*P* = 0.001).

### Enzyme-Linked Immunosorbent Assay

#### IL-10 Secretion in MG and HCs

Reduced IL-10 secretion significantly in B cells of patients with MG (*n* = 10, 0.20 ± 0.04) as compared to HCs (*n* = 10, 0.51 ± 0.09) (*P* = 0.001).

#### TGF-β1 Secretion in MG and HCs

Reduced TGF-β1 secretion significantly in B cells of patients with MG (*n* = 10, 0.56 ± 0.14) as compared to HC (*n* = 10, 0.95 ± 0.47) (*P* = 0.031).

### Overall Results

**Figure 3:**
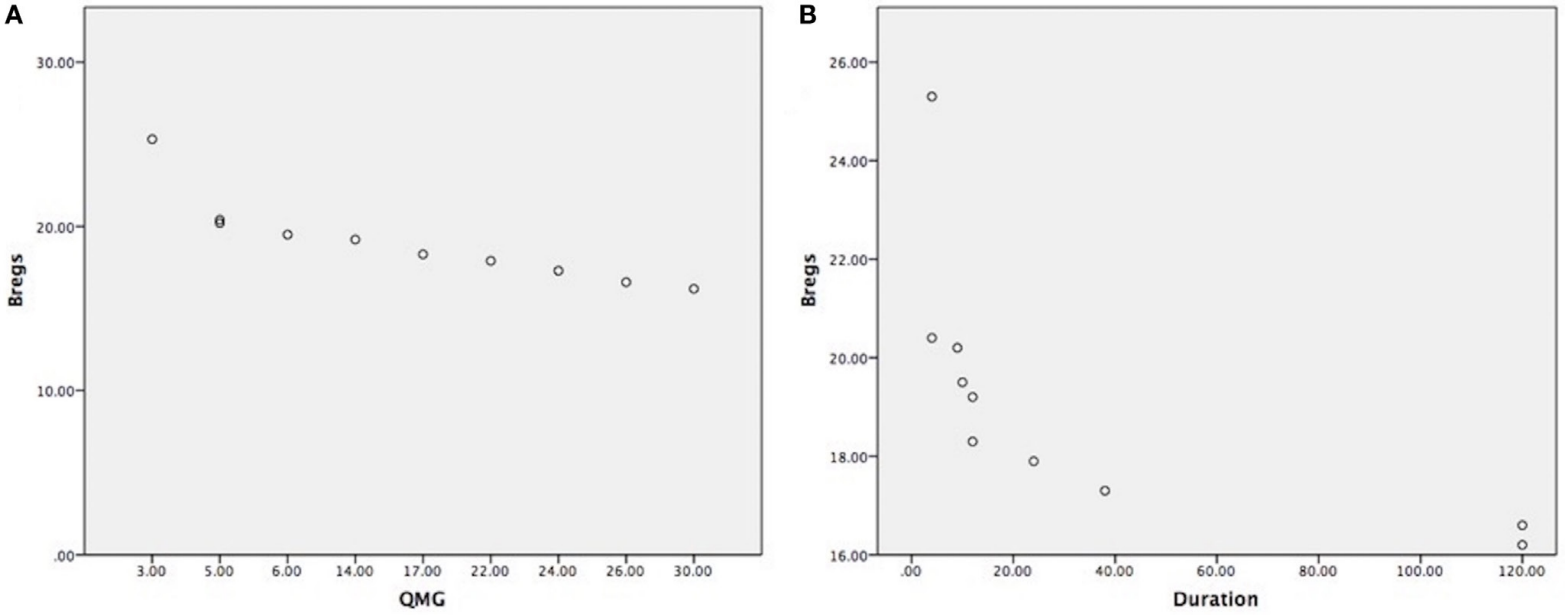
**(A)** Highly significant correlation between CD19^+^CD5^+^CD1d^+^ Bregs reduction in severe myasthenia gravis (MG) patients, as measured by quantitative myasthenia gravis scores (*r* = −0.844; *P* = 0.002); **(B)** statistically significant correlation between CD19^+^CD5^+^CD1d^+^ Bregs reduction and disease duration shown in months (*r* = −0.656; *P* = 0.040). (Note: Bregs were not affected by current medications taken, medications doses, and treatment duration as all the samples were collected in the morning before having medication and right before thymectomy was done. Patients were having the usual adult doses and continued medication when the patient was diagnosed for the first time with MG.).

Clinical outcome measures as per data collection (see Table 1), such as disease duration, medications were taken or thymectomy status of each patient. The reduction of CD19^+^CD5^+^CD1d^+^ Bregs was not affected by current medications taken as all the samples were collected in the morning and right before thymectomy was done. We have found that the correlation between the reduction of CD19^+^CD5^+^CD1d^+^ Bregs and severity of the disease status is highly significant as the reduction was more in severe patients as per MGFA clinical classification, as measured by QMG scores (*r* = −0.844; *P* = 0.002) (Figure 3A). We also have observed a statistically significant correlation between reduction of CD19^+^CD5^+^CD1d^+^ Bregs and disease duration (*r* = −0.656; *P* = 0.040) (Figure 3B).

## Discussions

In this research, we have established that a subset Bregs is related to the pathologic process of MG patients. The expression of CD19^+^CD5^+^CD1d^+^ Breg cells is reduced significantly in patients with MG (19.09 ± 2.61%) as compared to HCs (31.42 ± 5.55%) (*P* = 0.001). These deductions can be secondary to the pathological process of MG or a primary cause that leads to the initiation of autoreactive T and B cells. In order to distinguish the concept and apply the operational interpretation of Bregs in MG in addition to finding out the content *in vitro* expansion and suppression of effector T, B, and antigen-presenting cells, their antigen specificity and presence or absence in thymus abnormality tests could be conducted (32).

Furthermore, studies have found that the idea of Bregs has been greatly related with IL-10 productivity and hinted that a comparative study may be done among MG patients and HCs so that the expression of the B10 cells like IL-10 expression may be analyzed. The decreased secretion of IL-10 in MG may be consequential to the pathologic process of MG, and the aftereffect of these studies could contribute to an effective treatment progress of MG patients (19). So, through the ELISAs of human, IL-10 has been institutionalized here that the B cells are capable of producing both IL-10 in patients with MG (0.20 ± 0.04) but lesser as compared to HCs (0.51 ± 0.09) (*P* = 0.001). There has been a wide debate about the identification of Bregs and the role of IL-10 in autoimmune diseases. The CD5^+^CD1d^+^ Bregs identified in our study may be a minuscule subset of a larger population of IL-10 producing B cells or B10 cells, which as a whole are only identifiable by their capability to express IL-10.

In a previous study, the production of IL-10 was found to be higher in RA, primary Sjogren’s syndrome (SjS) and SLE pointing to B cell hyperactivity as the cause of these autoimmune diseases (41). *In vivo* dysregulation of IL-10 gene expression was studied in patients with RA, primary SjS, and SLE. Voluntary IL-10 production by PBMCs was measured by reverse transcription-polymerase chain reaction and ELISA in untreated patients with these diseases and in HCs. It had been found that the IL-10 production was dramatically higher in RA, primary SjS, and SLE patients than in HCs that could play a role in B cell extreme activity and in the progression of autoimmunity (42).

In SLE patients, a subset of Bregs defined as CD19^+^CD24^hi^CD38^hi^ with lesser IL-10 secretion and reduced suppressive function when matched with HCs. Nevertheless, recombinant IL-10 has been proposed as a therapeutic treatment for RA due to its efficacy in the mouse model collagen-induced arthritis (41).

Likewise, in this research, through the ELISAs of human, TGF-β1 has been found that the B cells are capable of producing both TGF-β1 in patients with MG (0.56 ± 0.14) but lesser as compared to HCs (0.95 ± 0.47) (*P* = 0.031). So, it has been observed that in patients with MG the CD19^+^CD5^+^CD1d^+^ Bregs are not only reduced in number as well as reductions in function by decreasing in cytokine production, e.g., IL-10 and TGF-β1. In this research, it has been also being noticed that the reduced number of cytokine production also differs from each other when to compare to HCs, e.g., IL-10 of MG is compared to HCs (*P* = 0.001); TGF-β1 of MG is compared to HCs (*P* = 0.031). It means the reduction of cytokine production in MG is more significant for IL-10 than TGF-β1, which also shows that the primary concern when further research on inducing cytokine production should be on IL-10, as reduced IL-10 production on MG is more significant than TGF-β1.

The study findings of the identification of the existence of Bregs and the significant reduction of the number of Bregs in MG as compared to HCs; the reduction of IL-10 and TGF-β1 secretion in patients with MG as compared to HC in this research could contribute to new B cell-mediated therapeutic strategies in future. If the CD19^+^CD5^+^CD1d^+^ Bregs in cell culture of MG patients could be induced whether to follow it may improve the function of MG disease status.

## Conclusion

In this research project, flow cytometry detection and ELISAs in peripheral blood films of 10 MG patients and 10 HCs were performed after isolation of B cells using B cell isolation kit II and MS column. The expression of CD19^+^CD5^+^CD1d^+^ Bregs was significantly decreased in patients with MG as compared to HC (*P* = 0.001). The IL-10 secretion was significantly reduced in patients with MG (*P* = 0.001); TGF-β1 secretion was also significantly reduced in patients with MG (*P* = 0.031) as compared to HC (see Table 2). From this research, it has been observed that the reduced number of B cells is able to produce IL-10 in MG patients but lesser than compared to HCs. Also, the reduction of Bregs in MG was significantly more with greater severity of the MG disease status according to the MGFA clinical classification. In addition, disease duration also had an effect on reduction of Bregs in MG but not as much as the severity of the disease.

**Table 2:** **CD19^+^CD5^+^CD1d^+^ Bregs expression and interleukin (IL)-10 and transforming growth factor (TGF)-β1 secretion in isolated B cells in patients with myasthenia gravis (MG) (*n* = 10) as compared to healthy controls (HCs) (*n* = 10)**.

Group	Bregs (%)	IL-10 (pg/ml)	TGF-β1 (pg/ml)
MG	19.09 (±2.61)	0.20 (±0.04)	0.56 (±0.14)
HC	31.42 (±5.55)	0.51 (±0.09)	0.95 (±0.47)

*Mean (±SD)*.

*CD19^+^CD5^+^CD1d^+^ Bregs expression and IL-10 and TGF-β1 secretion differ from MG with HCs*.

*P < 0.05 (compared with HCs, CD19^+^CD5^+^CD1d^+^ Bregs expression and IL-10 and TGF-β1 secretion in MG are significantly lower)*.

## Author Contributions

MK: concept and protocol development, data collection, sample collection, research plan, performing research, statistical analysis, and writing and review of the manuscript. H-YZ, JY, and QS: the manuscript review. Y-FW: supervision and critical review of the manuscript.

## Conflict of Interest Statement

The authors declare that the research was conducted in the absence of any commercial or financial relationships that could be construed as a potential conflict of interest.
