# Supplementary material for: Regulatory B Cells in Seropositive Myasthenia Gravis versus Healthy Controls
Source: Front Neurol. 2017 Feb 20;8:43. doi: 10.3389/fneur.2017.00043 (PMC5317198; doi:10.3389/fneur.2017.00043)

| **Group Statistics** | | | | | |
| --- | --- | --- | --- | --- | --- |
|  | Group | N | Mean | Std. Deviation | Std. Error Mean |
| IL10 | HC | 10 | .5126 | .08637 | .02731 |
|  | MG | 10 | .1992 | .03636 | .01150 |
| TGFBeta1 | HC | 10 | .9499 | .47357 | .14976 |
|  | MG | 10 | .5605 | .13664 | .04321 |
| B10 | HC | 10 | 31.4200 | 5.54673 | 1.75403 |
|  | MG | 10 | 19.0900 | 2.61383 | .82657 |

| **Independent Samples Test** | | | | | | | | | | |
| --- | --- | --- | --- | --- | --- | --- | --- | --- | --- | --- |
|  | | Levene's Test for Equality of Variances | | t-test for Equality of Means | | | | | | |
|  |  | F | Sig. | t | df | Sig. (2-tailed) | Mean Difference | Std. Error Difference | 95% Confidence Interval of the Difference | |
|  |  |  |  |  |  |  |  |  | Lower | Upper |
| IL10 | Equal variances assumed | 6.909 | .017 | 10.576 | 18 | .000 | .31340 | .02963 | .25114 | .37566 |
|  | Equal variances not assumed |  |  | 10.576 | 12.092 | .000 | .31340 | .02963 | .24889 | .37791 |
| TGFBeta1 | Equal variances assumed | 5.499 | .031 | 2.498 | 18 | .022 | .38940 | .15587 | .06194 | .71686 |
|  | Equal variances not assumed |  |  | 2.498 | 10.488 | .031 | .38940 | .15587 | .04429 | .73451 |
| B10 | Equal variances assumed | 5.308 | .033 | 6.359 | 18 | .000 | 12.33000 | 1.93903 | 8.25625 | 16.40375 |
|  | Equal variances not assumed |  |  | 6.359 | 12.809 | .000 | 12.33000 | 1.93903 | 8.13464 | 16.52536 |


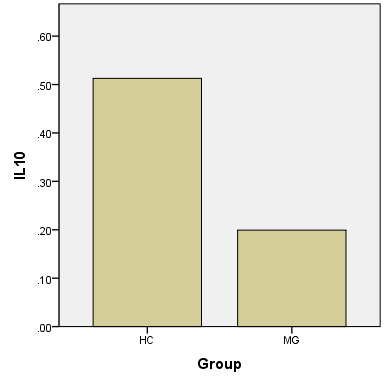


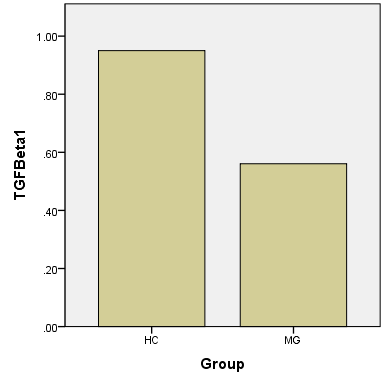


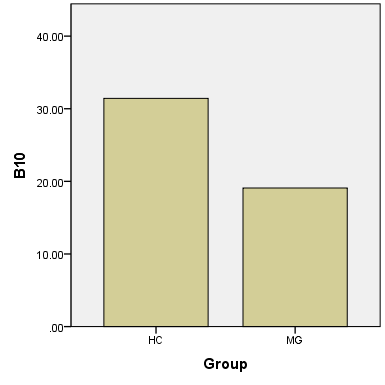

Supplement: Supplementary file 1 [file Data_Sheet_1.DOCX]
