# Supplementary material for: Regulatory B Cells in Seropositive Myasthenia Gravis versus Healthy Controls
Source: Front Neurol. 2017 Feb 20;8:43. doi: 10.3389/fneur.2017.00043 (PMC5317198; doi:10.3389/fneur.2017.00043)
Supplement: Supplementary file 2 [file Data_Sheet_2.DOC]

GET
  FILE='/Users/Dr_mdrezaulkarim/Desktop/QMG.sav'.
DATASET NAME DataSet1 WINDOW=FRONT.
DATASET ACTIVATE DataSet1.

SAVE OUTFILE='/Users/Dr_mdrezaulkarim/Desktop/QMG.sav'
  /COMPRESSED.
DATASET ACTIVATE DataSet1.

SAVE OUTFILE='/Users/Dr_mdrezaulkarim/Desktop/QMG.sav'
  /COMPRESSED.
EXECUTE.
DATASET ACTIVATE DataSet1.

SAVE OUTFILE='/Users/Dr_mdrezaulkarim/Desktop/QMG.sav'
  /COMPRESSED.
* Chart Builder.
GGRAPH
  /GRAPHDATASET NAME="graphdataset" VARIABLES=QMG Bregs MISSING=LISTWISE REPORTMISSING=NO
  /GRAPHSPEC SOURCE=INLINE.
BEGIN GPL
  SOURCE: s=userSource(id("graphdataset"))
  DATA: QMG=col(source(s), name("QMG"), unit.category())
  DATA: Bregs=col(source(s), name("Bregs"))
  GUIDE: axis(dim(1), label("QMG"))
  GUIDE: axis(dim(2), label("Bregs"))
  SCALE: linear(dim(2), include(0))
  ELEMENT: point(position(QMG*Bregs))
END GPL.


GGraph


Notes	
Output Created	03-OCT-2016 15:03:02	
Comments		
Input	Data	/Users/Dr_mdrezaulkarim/Desktop/QMG.sav	
	Active Dataset	DataSet1	
	Filter	<none>	
	Weight	<none>	
	Split File	<none>	
	N of Rows in Working Data File	10	
Syntax	GGRAPH
  /GRAPHDATASET NAME="graphdataset" VARIABLES=QMG Bregs MISSING=LISTWISE REPORTMISSING=NO
  /GRAPHSPEC SOURCE=INLINE.
BEGIN GPL
  SOURCE: s=userSource(id("graphdataset"))
  DATA: QMG=col(source(s), name("QMG"), unit.category())
  DATA: Bregs=col(source(s), name("Bregs"))
  GUIDE: axis(dim(1), label("QMG"))
  GUIDE: axis(dim(2), label("Bregs"))
  SCALE: linear(dim(2), include(0))
  ELEMENT: point(position(QMG*Bregs))
END GPL.	
Resources	Processor Time	00:00:00.98	
	Elapsed Time	00:00:01.00	


[DataSet1] /Users/Dr_mdrezaulkarim/Desktop/QMG.sav
