# Supplementary material for: Regulatory B Cells in Seropositive Myasthenia Gravis versus Healthy Controls
Source: Front Neurol. 2017 Feb 20;8:43. doi: 10.3389/fneur.2017.00043 (PMC5317198; doi:10.3389/fneur.2017.00043)
Supplement: Supplementary file 3 [file Data_Sheet_3.DOCX]

**Human IL-10 ELISA Kit Reference Value**
